# Associated medical conditions among 10-year-old children with oral clefts - a retrospective review across three cleft centres: Part 2

**DOI:** 10.1038/s41415-023-5975-6

**Published:** 2023-06-23

**Authors:** Samantha Gee, Maryam Ezzeldin, Jacob Curtis, Victoria J. Clark, Jacqueline Smallridge, Mechelle Collard

**Affiliations:** 114652808796092245944 Specialty Registrar and Honorary Clinical Teacher in Paediatric Dentistry, University Dental Hospital and School, Cardiff, United Kingdom; 895292775835815389303 Specialist and Honorary Clinical Teacher in Paediatric Dentistry, University Dental Hospital and School, Cardiff, United Kingdom; 228105136430651907935 Post Certificate of Completion of Training in Orthodontics, Morriston Hospital, Swansea, United Kingdom; 626298131440241827756 Consultant in Paediatric Dentistry, Birmingham Children´s Hospital, United Kingdom; 004156246257218274849 Consultant in Paediatric Dentistry, CleftNetEast, Cambridge University Hospitals, United Kingdom; 000378239598234040846 Consultant in Paediatric Dentistry, Morriston Hospital, Swansea, UK; Consultant and Honorary Senior Lecturer in Paediatric Dentistry, University Dental Hospital and School, Cardiff, United Kingdom

## Abstract

**Introduction ** In many cases, children with oral clefts present with accompanying medical conditions. These associated conditions can add complexity to the patient's dental management, both in terms of their treatment need and risk. Recognition and careful consideration of associated medical conditions is therefore crucial in providing safe and effective care for these patients.

**Aim** This paper is the second in a two-part three-centre series. It investigates the prevalence of medical conditions affecting cleft lip and/or palate patients attending three cleft units within the UK.

**Method** Retrospective review was undertaken within three cleft units: South Wales (SW), Cleft NET East (CNE) and West Midlands (WM). This was completed via assessment of the 10-year audit record appointment clinical notes for the year 2016/2017.

**Results** In total, 144 cases were reviewed (SW = 42; CNE = 52; WM = 50). Of these, 38.9% of patients (n = 56) had associated medical conditions recorded.

**Discussion** The review highlights the variety and impact of medical conditions affecting UK cleft patients providing insight into the consequent complexity of their dental care.

**Conclusion** An awareness of cleft lip and/or palate patients' associated medical conditions is important for all health care professionals involved in their care. Indeed, understanding of the patient's medical needs by multidisciplinary cleft teams is essential for effective planning and completion of holistic care. Involvement of specialists in paediatric dentistry sharing care with general dental practitioners is vital in providing appropriate oral health care and preventive support.

## Key points

- Children with oral clefts often present with associated medical conditions which must be identified and managed to minimise any impact on patient care, including cleft repairs and the treatment of dental pathology.
- A quarter of patients in this study were found to be affected by more than one type of medical condition. This is reflective of reports in the literature of the potential for cleft patients to present with multiple comorbidities and considerable medical and social needs.
- Dental practitioners working in primary care are paramount in the shared care of cleft patients. Their understanding of the patients' medical needs, liaison with medical specialists and support of appropriate dental preventive regimens is crucial in establishing desirable oral hygiene and successful outcomes for these patients.

## Introduction

Clefts of the lip and/or palate (CLP) are one of the most common congenital anomalies, reported to affect between 1 in 500-700 live births annually in the UK with an 'overall estimated incidence of cleft between 2010 and 2019 was 15.0 per 10,000 live births'.^1^^,^^2^^,^^3^ The presence of CLP can impact orofacial function leading to impaired suckling, altered hearing/deafness, speech impediments, malocclusion, facial deformity and psychological difficulties.^4^^,^^5^

The impact of the cleft on function may be in part due to the type or severity of the oral cleft and associated alveolar defect in which there may be great variance.^6^ The Cleft Registry and Audit Network has previously reported the most common cleft type is that affecting the palate only (44% of all oral clefts), followed by clefts of the lip, with and without inclusion of the underlying alveolus (24%), and those affecting both the lip and palate on one side only (22%). The most severe clefts are those affecting both the lip and palate bilaterally; these have been found to be less common, affecting 10% of all oral cleft patients.^6^

Importantly, though they can occur in isolation, orofacial clefts often present with other accompanying medical conditions, dental anomalies or congenital deformities that further complicate the patient's holistic needs and must be carefully considered in providing suitable care.^7^^,^^8^^,^^9^^,^^10^^,^^11^^,^^12^

Given the scope of these patients' medical and dental needs, they often require a wide variety of specialist health care teams to deliver coordinated multidisciplinary care.^1^^,^^8^ Paediatric dentists play an important role in this multidisciplinary team examining patients' dental needs and working towards maintainable dental hygiene and health.^4^^,^^13^ Their understanding of the patients' associated medical conditions is of great importance in safely achieving this goal.

This review looks to improve our insight into and understanding of the medical considerations required in providing holistic dental care.

## Aim

The primary aim of this study was to assess the prevalence of medical conditions currently affecting patients attending three cleft units in the UK.

This article builds on data and discussions presented in Part 1^12^ of this two-part, three-centre series, the focus of which was the investigation of dental anomalies affecting CLP patients.

Through discussion, we hope to achieve the following secondary aims:

- Describe the common medical conditions found in CLP patients
- Discuss the implications associated medical conditions can have on patients' dental risk and management in both primary care and specialist services
- Highlight the importance of holistic review of patients and regular prevention in supporting these patients.

## Method

This was a retrospective, three-centre, cross-sectional study in which data were collected from the clinical records and radiographs of patients attending audit 10-year record appointments within South Wales (SW), Cleft NET East (CNE) and West Midlands (WM) cleft units in 2016/2017. The clinical records and data reviewed were compiled by calibrated specialists in paediatric dentistry and form the basis of national audit data for CLP children in the UK.

Patients to be included were randomly selected from the three cleft unit's databases by use of a random number generator. All patients randomly selected, whose clinical records and multidisciplinary summaries could be accessed, were included, with a minimum of 40 patients per cleft unit specified.

Data were collected by four data collectors (one WM, one CNE and two SW) using an Excel spreadsheet. Data gathered and to be reviewed in this paper included patients' sex, type of cleft, medical conditions and whether they had undergone alveolar bone grafting. Additional data involving the presence of dental anomalies were collected and reviewed in Part 1.^12^

Medical conditions were subcategorised into the following groups:

- Cardiac - where congenital heart or ventral defects existed, such as, aortic stenosis or ventral septal defect
- Respiratory and ear, nose and throat (ENT) - where conditions impacted airway, olfactory or auditory systems, such as, asthma, chronic mucoid otitis media or hypertrophy of tonsils
- Neurological - where conditions had an effect on the patient's neurological function or learning capacity, such as, epilepsy, attention deficit disorder, autism, microcephaly
- Skeletal - where there were abnormalities of skeletal development, such as, scoliosis, spondyloepiphyseal dysplasia, talipes
- Syndrome and chromosomal abnormalities - where patients were diagnosed with such conditions including Pierre Robin, CHARGE syndrome or Jouberts syndrome.

Patients were included in multiple groups where the complexity of their medical condition demanded it.

## Results

**Fig. 1:**
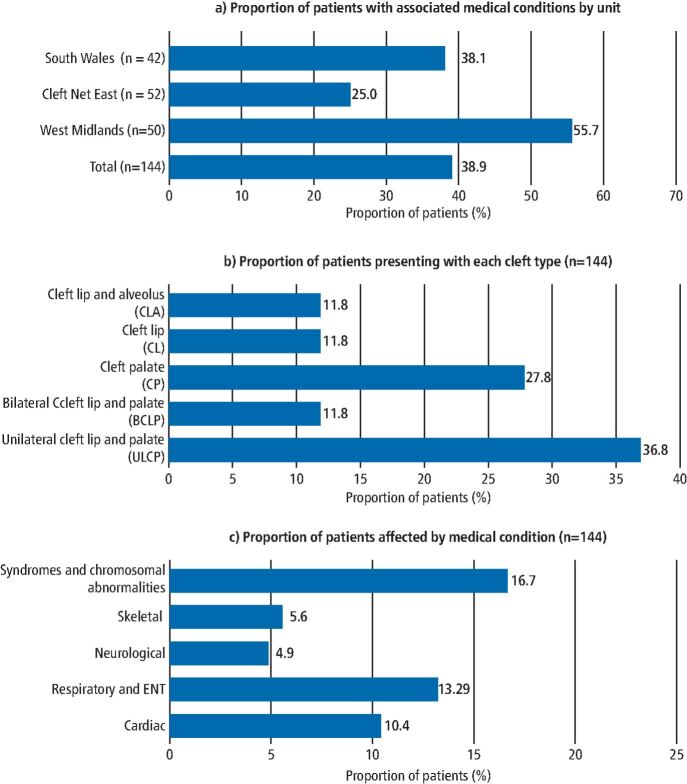
Cases of associated medical conditions described by: a) Cleft unit. b) Proportion of patients presenting with each cleft type. c) Proportion of patients affected by each type of medical condition. Note that some patients reported more than one of the medical conditions described

A total of 144 patients were reviewed (SW = 42; CNE = 52; WM = 50), with a split of 42% (n = 61) female and 58% (n = 83) male patients. Of the total patients, 38.9% (n = 56) had associated medical conditions recorded, though rates did vary across the cleft units, as shown in Figure 1.

All patients had a documented diagnosis of CLP, defined as either unilateral CLP (UCLP), bilateral CLP (BCLP), cleft palate (CP), cleft lip (CL) or cleft lip and alveolus (CLA). Within this review, patients were spread across all cleft types, with a minimum of 17 patients per cleft type. A higher proportion of patients had diagnoses of ULCP compared to other cleft types, as seen in Figure 1.

The number of medical conditions reported (n = 73) was greater than that of total patients affected (n = 56). This was a result of several patients presenting with conditions involving more than one medical subgroup.

Syndromes and chromosomal abnormalities were the most prevalent affecting 16.7% (n = 24) of cases. Lower rates of neurological and skeletal conditions were reported, at 4.9% (n = 7) and 5.6% (n = 8), respectively.

Almost three-quarters (73.2%; n = 41) of the 56 patients were found to have medical conditions represented under a single subcategory of medical condition. Also, 13 patients (23.2%) had medical conditions defined by two of the medical categories, while the remaining two patients were found to have more complex medical diagnoses involving three of the five subcategories.

**Fig. 2:**
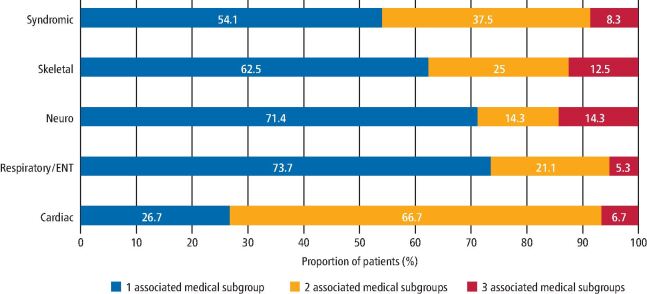
Graph showing the presentation of the medical subgroups in isolation and in combination with other types of medical conditions

There was variation in the rate with which the medical subgroups presented in combination with other conditions, as is shown in Figure 2. Respiratory and ENT, and neurological subgroups were more commonly recorded without the presence of other medical subgroups (73.7%; n = 14 and 72.4%; n = 5, respectively). Approximately half of the patients presenting with syndromes or other chromosomal abnormalities had other types of associated medical conditions (45.8%; n = 11).

Cardiac abnormalities more commonly presented in combination with other types of anomalies. Almost three-quarters (73.4%; n = 11) of the 15 patients with cardiac anomalies had other conditions diagnosed and three-fifths of them (60.0%; n = 9) had associated syndromes.

**Fig. 3:**
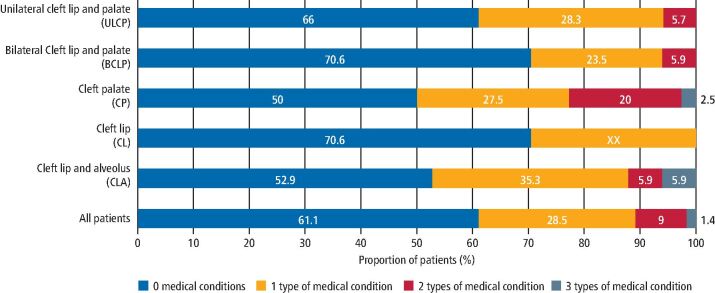
Proportion of patients within each cleft type presenting with associated medical conditions

The number and type of medical conditions reported also varied according to the patients' cleft type (Fig. 3). Approximately half of patients with CP or CLA presented without associated medical conditions: 50% (n = 20) and 52.9% (n = 9), respectively. This rate was lower than that seen with other cleft types reviewed, as seen in Figure 3.

Table 1 shows the difference in the types of medical conditions experienced by patients of each cleft type. Skeletal and neurological abnormalities presented more commonly in association with CLA, with rates reported at of 11.8% (n = 2) and 23.5% (n = 4) of CLA patients, respectively. Cardiac conditions, conversely, were found more commonly associated with CP patients, with 25% (n = 10) of CP cases affected.

**Table 1:** Percentage of patients by cleft type with each type of associated medical condition (n)

Medical condition	UCLP	BCLP	CP	CL	CLA
Cardiac	7.5 (4)	5.9 (1)	25 (10)*	0	0
Respiratory and ENT	15.1 (8)*	11.8 (2)	12.5 (5)	17.6 (3)*	5.9 (1)
Neurological	0	0	7.5 (3)	0	23.5 (4)*
Skeletal	3.8 (2)	0	7.5 (3)	5.9 (1)	11.8 (2)
Syndromic	13.2 (7)	17.6 (3)*	22.5 (9)*	5.9 (1)	23.5 (4)*
Total patients with an associated medical condition	34.0 (18)	29.4 (5)	50 (20)	29.4 (5)	47.1 (8)
Total patients with each cleft type	53	17	40	17	17
Key: * = conditions seen in greater than 15% of each cohort					

Similarly, there was variation in the number of medical subgroups associated with the different cleft types. Over one-fifth of patients with CP (22.5%; n = 9) presented with more than one type of medical condition. This was higher than that reported in other cleft types, as shown in Figure 3.

## Discussion

Embryological development of the head and neck requires successful coordination of an intricate cascade of transcription factors, signalling molecules and cell-to-cell interactions. Disturbances in this series can result in facial clefts, the most common of which are orofacial clefts, including CLP.^14^ The literature has previously reported approximately 70% of CLP patients are non-syndromic, their clefts being a result of disturbances without other cognitive or craniofacial anomalies.^1^^,^^15^^,^^16^ The findings of this study, however, found the proportion of non-syndromic clefts to be higher, at 83.3% (n = 120). It must be considered that there are likely a number of patients born with more severe syndromic traits who may not have survived until their tenth birthday and hence were not included in this study. Further prospective research from birth may be of benefit, both in detailing the occurrence and impact of this and in assessing the impact of other contributory factors, such as race and sex, which were not considered in this review.

Wyszynski *et al.* (2006) summarised a number of explanations for variations in the data associated with orofacial clefts.^17^ Suggested contributory factors included: the length of time after birth cases are examined; the variability in presentation of associated anomalies and definitions for such; the selection of patients; and true population differences and changes over time.^17^^,^^18^ These factors may explain not just the higher rate of non-syndromic clefts in this study, but also the reported variation in associated medical conditions between cleft units, with a higher rate of 55.7% seen at the WM site, compared to 38.1% and 25.0% at SW and CNE units, respectively. This is likely in part a result of the retrospective review of the different clinical record programmes and data input across the units.

In line with literature, this study showed the scope of comorbidities experienced by cleft patients. As Davis *et al.* (2021) reported, individuals with CLP are at increased risk of physical health issues persisting into adulthood.^19^ The medical conditions associated with orofacial clefts are not negligible; indeed, our study found respiratory and cardiac conditions were present in 13.2% and 10.4% of patients, respectively. These conditions have anaesthetic and/or surgical implications and must be identified and managed to minimise any impact on patient care, including cleft repairs and the treatment of dental pathology.^20^^,^^21^^,^^22^

The literature reports the prevalence of cardiac abnormalities among orofacial cleft patients to range from 2.32-30.7%.^21^^,^^23^^,^^24^^,^^25^^,^^26^ Our study found cardiac conditions to affect 10.4% of cleft patients, a rate within the previously identified range and comparable to that found by Barbosa *et al.* (9.5%) and Fakhim *et al.* (12%).^23^^,^^26^

As discussed by Azadgoli *et al.* (2020), the impact of cardiac conditions can be vast in cases where there are delays to cleft repair or increased rates of fistula post-operatively.^24^ Our data found cleft patients presenting with cardiac conditions commonly had further medical diagnoses complicating their care. This was the case for 73.4% (n = 11) of cardiac patients, a higher rate than that recorded with any other medical subgroup. It appears that a high number - three-fifths - of cardiac diagnoses in cleft patients are associated with syndromes or chromosomal abnormalities (60%; n = 9). Recognition of the association between orofacial clefts, cardiac conditions and syndromes or chromosomal abnormalities, ensuring careful examination of children with CLP for signs of heart disease, is important in reducing the morbidity of CLP surgery, making treatment more predictable and safer.^21^

Overall, one-quarter of patients (26.8%; n = 15) in this study were found to be affected by more than one type of medical condition. This is reflective of reports in the literature of the potential for cleft patients to present with multiple comorbidities and considerable medical and social needs.^19^

In this study, two patients were found to have complex medical diagnoses involving three of the five subcategories: one patient with chromosomal abnormality, brain deformity, microcephaly and epilepsy, among other conditions pertaining to neurological, skeletal and syndromic categories; the second, a patient with Pierre Robin sequence, ventricular septal defect and asthma defined under cardiac, respiratory and syndromic categories. These cases demonstrate the wide variety and severity of medical conditions that can affect cleft patients and the importance therefore in multidisciplinary management to manage their holistic needs.^1^^,^^8^^,^^27^

Our data found there to be variation both in the number and type of medical conditions experienced by different cleft types. Though not statistically significant, higher rates of associated medical conditions were seen within CP (50%; n = 20) and CLA (47.1; n = 8) patient groups compared to other cleft types. Similarly, a higher number of medical conditions/subgroups were seen associated with CP than other cleft types. Over one-fifth of CP patients (22.5%; n = 9) presented with more than one type of medical condition. This contrasts with findings regarding the number and variety of dental anomalies associated with the different cleft types reported in Part 1,^12^where CP patients were found to have the lowest mean number of teeth affected. Though further research is required to assess whether these variations are statistically or clinically significant, it highlights the different experiences of patients with CLP and the need for individualised plans and care.

### Implications for dentists working in primary care

As discussed in Part 1,^12^cleft patients have a higher prevalence of dental anomalies including tooth agenesis, ectopic impactions and lateral incisors of poor morphology. As we have highlighted in this paper, these patients also often present with medical comorbidities that can vary by cleft type and severity.

In order to ensure their complex medical, dental and social needs are met, it is recommended that all patients with craniofacial anomalies, including those with orofacial clefts or CLP, are managed by multidisciplinary teams.^8^^,^^28^^,^^29^ Paediatric dentists are an important part of this team, not only due to the high rate of dental anomalies experienced by these patients as discussed in Part 1,^12^ but in minimising the impact of dental anomalies and disease on accompanying medical and surgical needs.^4^^,^^9^^,^^10^^,^^11^

To this end, an awareness of patients' medical conditions is paramount to the safe planning of dental treatment. Dental practitioners undertaking treatment for patients with cardiac conditions should liaise with specialist cardiac colleagues and implement guidance set out by the Scottish Dental Clinical Effectiveness Programme to minimise the risk of infective endocarditis and safely manage patients' anticoagulant or antiplatelet therapies during dental treatment.^9^^,^^30^^,^^31^

Similarly, in planning dental treatment for patients with respiratory, ENT, neurological and skeletal anomalies, consideration must be given to the potential need for additional airway support and the impact these conditions may have on patient positioning, suitability for treatment under general anaesthetic and possible need for extended inpatient reviews following treatment.^9^^,^^10^^,^^11^^,^^20^^,^^22^

Patients presenting with syndromes and chromosomal abnormalities can have great variation in their features and experience, hence an individualised approach liaising with their appropriate specialist health care professionals in a multidisciplinary approach as discussed is recommended for all.^8^

Within the UK, there are 12 cleft services operating through 17 associated cleft units, and a further service with three cleft units providing care for CLP patients in the Republic of Ireland. These units deliver multidisciplinary specialist support and clinical care to patients with CLP from diagnosis through to adulthood.^32^^,^^33^ Patients can access services, returning for further advice at any age. Practitioners in primary care should ensure patients are aware of how to access treatment and liaise appropriately with local cleft units to coordinate patient care where the need arises.^19^

Following a patient's assessment within a cleft unit, relevant primary care professionals receive written documentation of the team's findings and management plan with detail of support required in primary care.^8^ Dental practitioners working in primary care are paramount in the shared care of cleft patients. Their role in supporting dental preventive regimens, acclimatising patients to the dental environment and treatment, and establishing desirable oral hygiene, health and habits in these patients cannot be underestimated. Their promotion of patients' oral health is of great importance in the preparation for and success of reparative surgery, dental treatment and management of accompanying comorbidities.^4^^,^^13^

## Conclusion

This article gives insight into the medical conditions that may affect CLP patients. A considerable proportion of cleft patients will experience one or more comorbidity. Recognition and understanding of patients' medical conditions, needs and impact is of great importance in safely managing patients' care.

Together with Part 1,^12^ this three-centre study highlights the importance of multidisciplinary assessment and treatment in ensuring patients receive appropriate access to the required specialist-led care.

There are a multitude of dental anomalies and medical conditions that can affect CLP patients. Intensive preventive oral hygiene regimes supported by primary care practitioners is thus a crucial part of their holistic care optimising oral and general health.
